# Identification of potential inhibitors for drug-resistant EGFR mutations in non-small cell lung cancer using whole exome sequencing data

**DOI:** 10.3389/fphar.2024.1428158

**Published:** 2024-07-25

**Authors:** Nagasundaram Nagarajan, Chittibabu Guda

**Affiliations:** ^1^ Department of Genetics, Cell Biology and Anatomy, University of Nebraska Medical Center, Omaha, NE, United States; ^2^ Center for Biomedical Informatics Research and Innovation, University of Nebraska Medical Center, Omaha, NE, United States

**Keywords:** NSCLC, EGFR, cancer drug resistance, afatinib, osimertinib, precision drug discovery

## Abstract

Epidermal growth factor receptor (EGFR) gene mutations are prevalent in about 50% of lung adenocarcinoma patients. Highly effective tyrosine kinase inhibitors (TKIs) targeting the EGFR protein have revolutionized treatment for the prevalent and aggressive lung malignancy. However, the emergence of new EGFR mutations and the rapid development of additional drug resistance mechanisms pose substantial challenge to the effective treatment of NSCLC. To investigate the underlying causes of drug resistance, we utilized next-generation sequencing data to analyse the genetic alterations in different tumor genomic states under the pressure of drug selection. This study involved a comprehensive analysis of whole exome sequencing data (WES) from NSCLC patients before and after treatment with afatinib and osimertinib with a goal to identify drug resistance mutations from the post-treatment WES data. We identified five EGFR single-point mutations (L718A, G724E, G724K, K745L, V851D) and one double mutation (T790M/L858R) associated with drug resistance. Through molecular docking, we observed that mutations, G724E, K745L, V851D, and T790M/L858R, have negatively affected the binding affinity with the FDA-approved drugs. Further, molecular dynamic simulations revealed the detrimental impact of these mutations on the binding efficacy. Finally, we conducted virtual screening against structurally similar compounds to afatinib and osimertinib and identified three compounds (CID 71496460, 73292362, and 73292545) that showed the potential to selectively inhibit EGFR despite the drug-resistance mutations. The WES-based study provides additional insight to understand the drug resistance mechanisms driven by tumor mutations and helps develop potential lead compounds to inhibit EGFR in the presence of drug resistance mutations.

## 1 Introduction

Lung cancer, ranking amongst the most prevalent and deadliest malignancies worldwide, poses a significant threat to human health and quality of life. In 2020 alone, over 2.2 million new cases were identified and about 1.80 million people succumbed to this disease (Sung et al., 2021). The 5-year survival rates for lung cancer are notably low, with 17% for men and 24% for women (Bray et al., 2018). The increased expression of epidermal growth factor receptor (EGFR) has been associated with the development of various human cancers, including non-small cell lung cancer (NSCLC) (Ohsaki et al., 2000; Inamura et al., 2010). EGFR is a transmembrane receptor kinase that is expressed in epithelial, mesenchymal, and neurogenic tissues. Several studies have shown that higher EGFR expression in NSCLC is correlated with poorer survival rates (Scagliotti et al., 2004), increased incidence of lymph node metastasis (Fang et al., 2014), and diminished response to chemotherapy (Ogawa et al., 1993; Swinson et al., 2004). First-generation EGFR tyrosine kinase inhibitors (TKIs) such as erlotinib, gefitinib, icotinib, and lapatinib have been widely used to inhibit EGFR activity, reversibly and ATP-competitively. These EGFR TKIs have demonstrated enhanced cytotoxic effects on mutated forms of EGFR (Guardiola et al., 2019).

However, despite the initial efficacy of first-generation EGFR TKIs, nearly all NSCLC patients eventually develop resistance to these drugs within 10–14 months, primarily due to the emergence of the EGFR mutation, T790M (Wu and Shih, 2018). Second-generation EGFR TKIs have been developed to overcome this resistance with a more potent inhibitory effect on EGFR (Guardiola et al., 2019). Second generation agents such as afatinib, neratinib, and dacomitinib have demonstrated superior anticancer activity compared to their first-generation counterparts (Guardiola et al., 2019). In response to the growing resistance challenge, FDA has also approved osimertinib, a third-generation irreversible EGFR TKI, for treating patients who have developed resistance to both first- and second-generation drugs. In addition, Osimertinib was also approved as a first-line therapy for patients with EGFR mutation-positive tumors. Despite the substantial progress made with third-generation TKIs, patients continue to acquire resistance and fail to respond to these inhibitors. Over time, all patients eventually develop resistance, indicating that acquired resistance mechanisms diminish the efficacy of these medications. Despite the significant therapeutic advancements and improved understanding of the genetic foundations, developing resistance to EGFR TKIs remains inevitable, leading to disease progression (Westover et al., 2018; Del Re et al., 2019). This is partly due to the genetic heterogeneity among the NSCLC patients. Therefore, gaining insights into the unique genetic makeup of individuals can pave the way for precision treatment approaches tailored to a patient’s mutational profile.

Genomic sequencing has revolutionized precision drug discovery by offering valuable insights into the mutational profiles of the genetically heterogeneous diseases (Strianese et al., 2020). Recent advancements in sequencing technology have made it feasible to sequence the entire tumor genome or specific regions of interest, quickly and affordably, to enable the monitoring of acquired mutations linked to drug resistance throughout the cancer life cycle. This paradigm shift in genome sequencing technologies has fuelled the development of personalized medicine approaches by empowering researchers to pinpoint genetic and drug-resistant mutations linked to a particular disease such as cancer.

**FIGURE 1:**
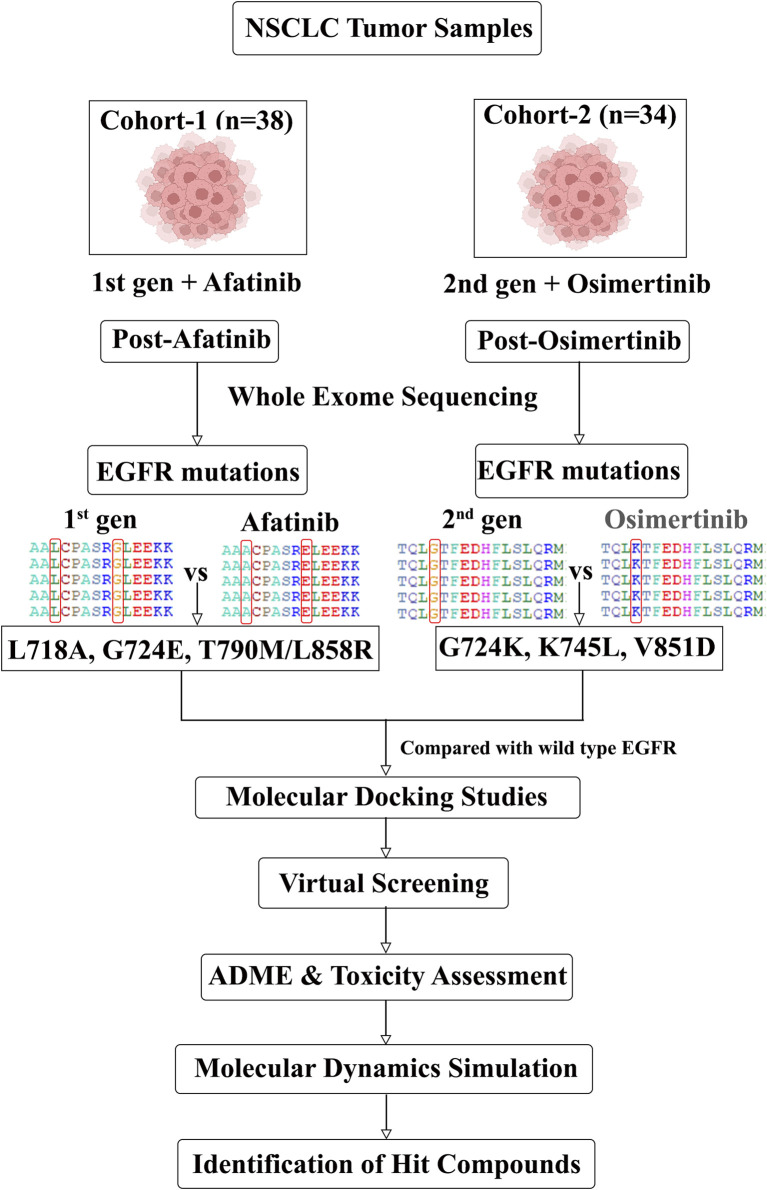
Schematic showing the *in silico* workflow from mutation detection to drug discovery.

In this study, we examined two genomic cohorts of NSCLC patients (SRA IDs PRJEB21459 and PRJNA616048/dbGaP: phs002001) who exhibited resistance to the second and third-generation drugs, afatinib and osimertinib, respectively. By analyzing the whole exome sequences (WES) of NSCLC patients before and after the development of drug resistance, we identified EGFR mutations associated with this resistance for each drug. Subsequently, we conducted molecular modeling and docking studies to assess the binding affinity between the mutant EGFR and the FDA-approved drugs (afatinib and osimertinib) used for the treatment. Following that, we performed virtual screening to identify promising structurally-similar lead compounds capable of inhibiting EGFR despite the presence of drug-resistance mutations. To gain further insights, molecular dynamic simulations were carried out to evaluate the binding efficacy of the screened compounds with the drug-resistant mutant structures of EGFR. The overall workflow of our approach is depicted in Figure 1.

## 2 Methodology

### 2.1 Data collection

In this study, we utilized WES data obtained from the tumor samples of two NSCLC patient cohorts. These data were collected from the SRA (Sayers et al., 2022) and dbGAP (Wong et al., 2017) databases. The first cohort contains 38 patients who received the initial treatment with Erlotinib/Gefitinib (SRA ID PRJEB21459) (van der Wekken et al., 2017) and subsequently were treated with the second-generation drug, Afatinib. The second cohort of 34 patients were treated with the same first and second-generation drugs as the first cohort but also received an additional treatment with a third-generation drug, osimertinib (PRJNA616048/dbGaP: phs002001). The crystal structure of the EGFR protein (PDB ID: 3VJO) was obtained from the RCSB-PDB database, and using PyMOL software (PyMoL, 2010), water molecules and other bound molecules were removed. Mutant models were constructed using SPDBV 4.10 software (Guex et al., 1997).

### 2.2 Whole exome sequencing data analysis

We employed *Nextflow-Sarek 3.1.2* to analyse whole exome sequencing (WES) data, which is a comprehensive workflow designed for quality control, germline and somatic variant detection, and annotation using the recommended best practices in the field (Garcia et al., 2020). During the pre-processing step, sequencing reads were aligned to the human reference genome (GRCh38/hg38) using *BWA-MEM* (Li et al., 2013) and deduplication and recalibration were carried out using *GATK* (McKenna et al., 2010). Since our objective was to identify somatic variants in patients with drug resistance, we utilized *GATK4 Mutect2* (Cibulskis et al., 2013) and *Strelka2* (Kim et al., 2018) to detect somatic single-base mutations (SSM) and small somatic insertion/deletion mutations (SIM). We employed *Manta* to detect somatic structural variants, including copy-number variation, ploidy, and sample purity (Chen et al., 2016). In order to evaluate the potential functional impacts of the identified variants, we annotated them using *snpEff* (Cingolani et al., 2012) and *VEP* (McLaren et al., 2016) The *nf-Sarek* workflow generates comprehensive quality control metrics, including *FastQC* (Li et al., 2009; Li, 2011; Okonechnikov et al., 2016) and *VCFtools* (Danecek et al., 2011), which are aggregated and visualized across samples using *MultiQC* (Ewels et al., 2016).

### 2.3 Preparation of EGFR wild-type (WT) and mutant-type structures

We employed the *Schrodinger suite’s* prime module and protein preparation wizard to ensure the integrity of the EGFR wild-type (WT) and mutant structures. This step involved the removal of artifacts such as incorrect bond orders, missing hydrogen atoms, misaligned groups, erroneous charge states, and the missing side chains (Schrodinger Release 2020-1. Protein Preparation Wizard; Schrodinger Release 2020-1. Prime). Additionally, restrained energy minimization was performed to alleviate strained bonds, angles, and steric hindrance, allowing heavy atoms to move within a range of 0.3 Å.

### 2.4 Preparation of compounds for molecular docking and virtual screening

The three-dimensional structures of FDA-approved EGFR inhibitors, namely, afatinib (CID:10184653) and osimertinib (CID:71496458), were obtained from the PubChem database (Kim et al., 2023). Using their structure information, 3505 and 3880 compounds that are structurally similar to afatinib and osimertinib, respectively, were retrieved from the PubChem and DrugBank (Wishart et al., 2018) databases. The *Maestro* tool was employed to prepare all the compounds for further analysis. *Ligprep*, a software tool, was used to generate 2D or 3D structures and corresponding low-energy 3D structures of both the approved EGFR inhibitors as well as the retrieved structurally similar compounds to make them ready for docking using the *Glide* program. Default parameters were used, except for the chirality feature, for which all combinations of chirality were considered. Subsequently, tautomer generation, desalting, and adjustment of probable ionization states at pH 7 ± 2 were performed (Zagaliotis et al., 2022). The *S. suite’s* Epik module, an integrated tool, was utilized to predict the ionization states of the molecules (Rajamanickam et al., 2022).

### 2.5 Molecular docking

The Schrodinger suite’s *Glide* module was employed to conduct site-specific molecular docking of FDA-approved EGFR inhibitors and virtually screened compounds against both EGFR WT and mutant targets. Receptor grid preparation was performed using the *Glide* tool with default parameters, including a partial charge cutoff of 0.25 and van der Waals radius scaling factor of 1.0 (Schrodinger Release 2020-1, Glide). For the screening process, *Glide* was utilized at extra precision (XP), which signifies a clear correlation between high-quality poses and favorable scores, ensuring an accurate evaluation of the libraries.

### 2.6 ADME and toxicity analysis

*QikProp* is a specialized tool designed to rapidly predict the ADME (absorption, distribution, metabolism, and excretion) properties of compounds with high accuracy (Schrodinger Release 2020-1. QikProp). It provides predictions for key physicochemical descriptors and pharmaceutical properties of organic molecules, either for individual compounds or in a batch mode. The predicted ADME properties encompass various parameters such as molecular weight, number of H-bond acceptors and donors, indicated octanol/water partition coefficient (MLogP), total polar surface area (TPSA), Lipinski’s rule of five (drug-likeness), Rat LD50, and hepatotoxicity. These predictions are crucial for assessing the pharmacokinetic and toxicological profiles of the compounds in the early drug discovery and development stages.

### 2.7 Molecular dynamics simulation

To assess the structural stability of the docked complexes involving WT and mutant EGFR targets with FDA-approved EGFR inhibitors (Afatinib and Osimertinib) as well as the virtually screened compounds, a 50 ns(nano seconds) molecular dynamics (MD) simulation was performed using *GROMACS gromacs/2021.1* software (Abraham et al., 2015). The topological parameters of the WT and mutant EGFR inhibitors were generated using the *PRODRG* web server (Schuttelkopf and van Aalten, 2004). The WT and mutant structures were solvated in cubic boxes using the SPC (single point charge) water model, ensuring a minimum distance of 1 nm from the box edges. For the structures complexed with ligand molecules, a similar solvation procedure was followed, with water molecules positioned at a 1 nm distance from the box borders, and counter ions (sodium and chloride) were added to neutralize the system. The structures underwent two rounds of energy minimization using the steepest descent technique followed by the conjugate gradient algorithm for 5000 steps to relax the system. Subsequently, the minimized systems were equilibrated under position-restrained ensemble conditions (NVT and NPT) at 300 K for 50,000 picoseconds (ps). Berendsen’s weak coupling was employed to maintain a constant pressure of 1 bar, and the Parrinello-Rahman approach (Martonak et al., 2003) was used to control the temperature at 300 K. The calculation of electrostatic interactions utilized the Fast Particle-Mesh Ewald electrostatics (PME) method (Hess et al., 1997) and a 50 ns long-range production MD run was conducted for both WT and mutant systems for each complex. To analyze the MD trajectories, *GROMACS* utility tools such as *g_rmsd, g_hbond, g_mindist, and g_sasa* were employed to examine the RMSD (Root Mean Square Deviation), number of hydrogen bonds, minimum distance between the protein and ligand, and solvent-accessible surface area of the protein, respectively. These analyses provided insights into the stability and dynamic behavior of the studied complexes during the MD simulation.

## 3 Results

### 3.1 Whole exome sequencing analysis identifies single- and double-point mutations

WES data analysis was conducted to identify EGFR drug resistance mutations in NSCLC patient samples. A total of 72 patients containing pre- and post-treatment WES data with osimertinib and afatinib were analyzed using *Nextflow Sarek 3.1.2*. In the post-treatment samples, five single-point mutations and one double mutation were identified, as shown in Table 1. Mutations L718A, G724E, T790M/L858R were found in post-afatinib treatment samples, while mutations G724K, K745L, and V851D were identified in post-osimertinib treated samples. All of these mutations except V851D have been previously reported as drug resistant on EGFR in various studies (Lu et al., 2018; Lu et al., 2020; Du et al., 2021; He et al., 2021). Further analysis of the binding modes of mutant EGFR variants with drug compounds will provide insights into the mechanism of drug resistance.

**TABLE 1:** Detected EGFR mutations in the samples collected after afatinib and osimertinib treatments.

EGFR mutations present in the post-afatinib treatment samples (n = 38)		EGFR mutations present in the post-osimertinib treatment samples (n = 34)	
Identified EGFR mutations	Number of samples with mutation(s)	Identified EGFR mutations	Number of samples with mutation(s)
L718A	2	G724K	1
G724E	1	K745L	1
T790M/L858R	16	V851D	13

### 3.2 Ligand binding affinity and hydrogen bonding pattern differ between WT and mutant EGFR variants

Comparative docking of EGFR WT and mutant structures with the FDA-approved drugs, osimertinib and afatinib, was performed using the *Glide* module of the *Schrodinger suite* (Schrodinger Release, Glide; Schrodinger, 2020; Halgren et al., 2004). The binding affinity of the ligands was evaluated based on the *Glide XP* Gscore, which was used to rank the poses of the ligands (Table 2). Previous studies have identified the drug-binding residues of EGFR at VAL726, ALA743, ILE744, LYS745, MET766, LUE789, THR790, GLN791, LEU792, MET793, GLY796, CYS797, ASP800, LEU844, and THR854 (Kashima et al., 2020). We observed that both afatinib and osimertinib bind to the EGFR mutant structures in a slightly different orientation than to the WT EGFR. For afatinib docking with the WT and mutant L718A, G724E, and T790M/L858R structures, the binding energies were −8.378, −8.6434, −7.8765, and −7.857 kcal/mol, respectively. Similarly, the binding energies for osimertinib docking with the WT, G724K, K745L, and V851D structures were −8.376, −8.314, −7.887, and −7.378 kcal/mol, respectively. The lower the binding energy, the higher the binding affinity, and *vice versa*. The binding energy between the mutant structure L718A and afatinib is almost similar to the binding energy of WT with afatinib, but the other mutant structures (G724E and T790M/L858R) obtained higher binding energy compared to the WT-Afatinib complex. Similarly, the binding energy between the mutant structure G724K and osimertinib is almost similar to that of the WT with osimertinib, but the other mutant structures (K745L and V851D) obtained higher binding energies compared to the WT-osimertinib complex. Because G724E, T790M/L858R, K745L, and V851D mutant structures obtained higher binding energies, that negatively affects their binding affinity with the corresponding drugs. Hence, these mutant structures were considered for further virtual screening studies taking into account their drug interaction patterns and dynamics.

**TABLE 2:** Molecular docking analysis between WT and Mutant EGFR with FDA-approved drugs: Afatinib and osimertinib. Table showing the respective targets, binding energy, hydrogen bonds formed between target and ligand, and the amino acids involved in the hydrogen bond formations.

FDA-approved drugs	Drug targets	XP gscore (Kcal/mol)	Number of hydrogen bonds between target and ligand	Amino acids in hydrogen bonding
Afatinib	WT EGFR	−8.378	2	LEU718, MET793
	L718A	−8.643	2	MET793
	G724E	−7.876	1	ASP855
	T790M/L858R	−7.857	1	MET793
Osimertinib	WT EGFR	−8.376	3	LEU718, MET793, CYS797
	G724K	−8.314	3	LEU718, MET793, CYS797
	K745L	−7.887	2	LEU718, MET793
	V851D	−7.378	1	CYS797

The interaction patterns based on hydrogen bonding between WT EGFR and its mutant’s post-treatment with afatinib and osimertinib were examined (Supplementary Figures S1, S2). Osimertinib formed three hydrogen bonds with both WT and G724K mutant EGFR structures, involving the same amino acid residues: LEU718, MET793, and CYS797 (Supplementary Figures S1A, S1B). On the other hand, this drug established two hydrogen bonds involving residues LEU718 and MET793 (Supplementary Figure S1C) with K745L mutant structure and only one hydrogen bond with ASP855 in the V851D mutant structure (Supplementary Figure S1D). With afatinib, two hydrogen bonds were established each in WT and L718A mutant both involving MET793 ( Supplementary Figures S2A, S2B). However, afatinib formed only one hydrogen bond each in G724E mutant (with MET793) and T790M/L858R double mutant (with MET793) structures (Supplementary Figures S2C, S2D). The mutant structures K745L, V851D, G724E, and T790M/L858R exhibited high binding energies and fewer hydrogen bonds with their corresponding drugs compared to the wild type (WT). Consequently, these four mutant structures G724E, T790M/L858R, K745L, and V851D with corresponding docked drugs were selected for further molecular dynamics simulations.

### 3.3 Comparison of binding efficacy, stability, and conformational dynamics between WT and mutant complexes with drugs using MD simulations

The primary objective of the extended molecular dynamics (MD) simulations was to investigate the comparative binding efficacy between the docked mutant complexes and WT complexes. A 50 ns MD simulation was performed for the following six protein-ligand complexes that include three for each drug: WT EGFR-Osimertinib, K745L-Osimertinib, V851D-Osimertinib, WT EGFR-Afatinib, T790M/L858R-Afatinib, and G724E-Afatinib. The GROMOS 53a6 force field in GROMACS was employed for energy minimization. Our analysis was focused on the backbone root-mean-square deviation (RMSD), hydrogen bonds, minimum distance, and the solvent-accessible surface area. For the complexes of WT EGFR, K745L, and V851D with osimertinib, the backbone RMSD analysis (Supplementary Figure S3A) revealed that WT EGFR exhibited a lower deviation pattern (∼0.25 nm) compared to the mutant structures, K745L (∼0.3 nm), and V851D (∼0.35 nm). Similarly, for the complexes of afatinib (Supplementary Figure S3B), WT EGFR displayed a lower deviation pattern (∼0.25 nm) compared to the mutant structure, G724E (∼0.35 nm), and double mutant, T790M/L858R (∼0.35 nm). Higher deviations in RMSD may impact the structural stability of the protein and subsequently lower the binding efficacy of the drugs.

**FIGURE 2:**
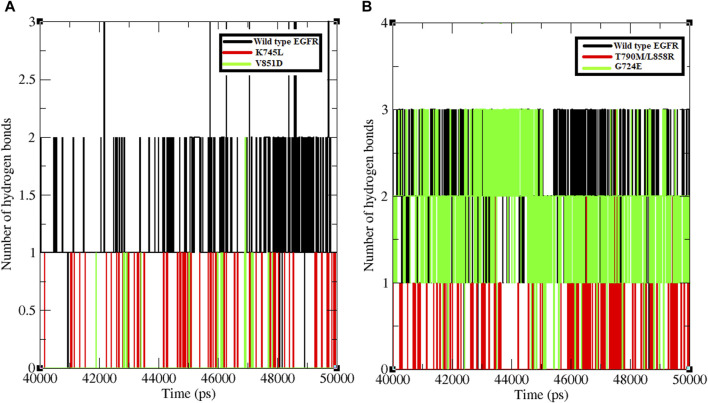
Number of hydrogen bonds formed between protein and ligand. **(A)** Number of hydrogen bonds formed between WT and mutant EGFR with drug osimertinib. **(B)** Number of hydrogen bonds formed between WT and mutant EGFR with drug afatinib.

Hydrogen bond formation between EGFR WT and mutant structures with osimertinib and afatinib was also analysed. The number of hydrogen bonds formed between osimertinib and WT EGFR, K745L, and V851D structures during the last 10 ns of the simulation was examined (Figure 2). WT EGFR in complex with osimertinib formed 1–3 hydrogen bonds, whereas the mutant complexes, K745L-osimertinib and V851D-osimertinib, formed fewer hydrogen bonds ranging from 0–1 and 0–2, respectively. Likewise, the number of hydrogen bonds between afatinib and WT EGFR or G724E complexes ranged from 1–3, while those between afatinib and T790M/L858R complex were fewer (0–2), as shown in Figure 2B. A lower number of hydrogen bonds may impact the stability of the protein-drug complex.

**FIGURE 3:**
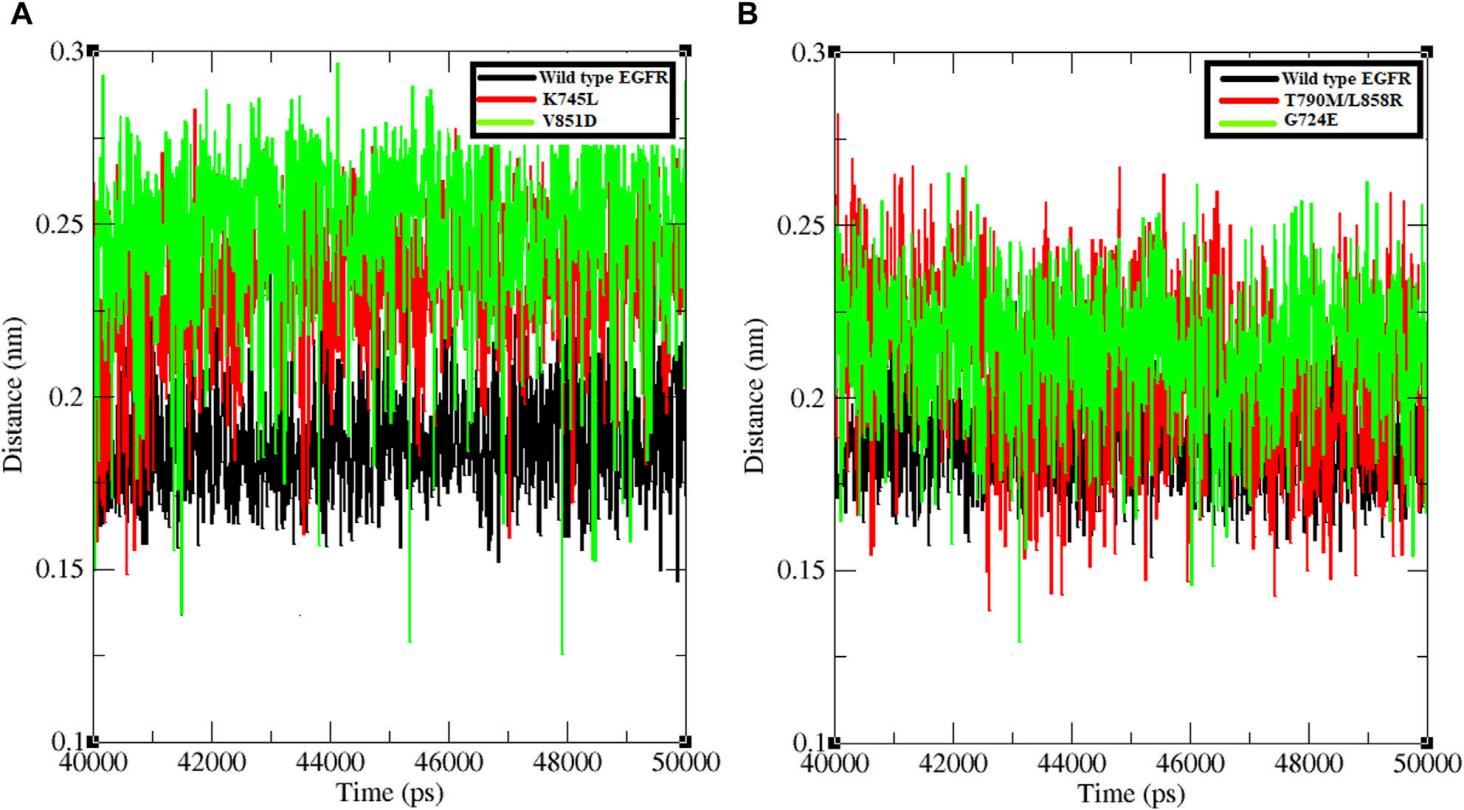
Minimum distance formed between protein and ligand. **(A)** Minimum distance formed between WT and mutant EGFR with drug osimertinib. **(B)** Minimum distance formed between WT and mutant EGFR with drug afatinib.

The minimum distance between WT EGFR, K745L, and V851D with osimertinib was analysed during the last 10 ns of the simulation period (Figure 3A). In the WT EGFR-Osimertinib complex, this distance was maintained at approximately 0.15–0.20 nm. However, the mutant complexes, K745L-Osimertinib and V851D-Osimertinib, exhibited higher distances of around 0.15–0.27 and 0.15–0.30 nm, respectively. Similar measurements in the afatinib associated complexes with WT EGFR, G724E, and T790M/L858R structures recorded these distances around 0.15–0.22, 0.15–0.25, and 0.15–0.27 nm, respectively. A higher distance between the components may impact the formation of non-bonded interactions within the complexes.

Further, SASA analysis was performed to compare the solvent accessible surface area in response to the overall protein conformational changes in the complex structures of EGFR WT and mutants with the two drugs. In the osimertinib-associated complexes, WT EGFR had a higher accessible area of approximately 170 nm^2^ than the mutants, K745L (∼155 nm^2^) and V851D (∼150 nm^2^) (Supplementary Figure S4A). Similarly, with afatinib complexes, the WT EGFR had a higher accessible area of approximately 160 nm^2^ than the mutants, G724E (∼152 nm^2^) and T790M/L858R (∼155 nm^2^) (Supplementary Figure S4B). A lower accessible surface area suggests fewer possibilities for interactions with other molecules.

### 3.4 Virtual screening identifies potential inhibitors of mutant EGFR structures

**FIGURE 4:**
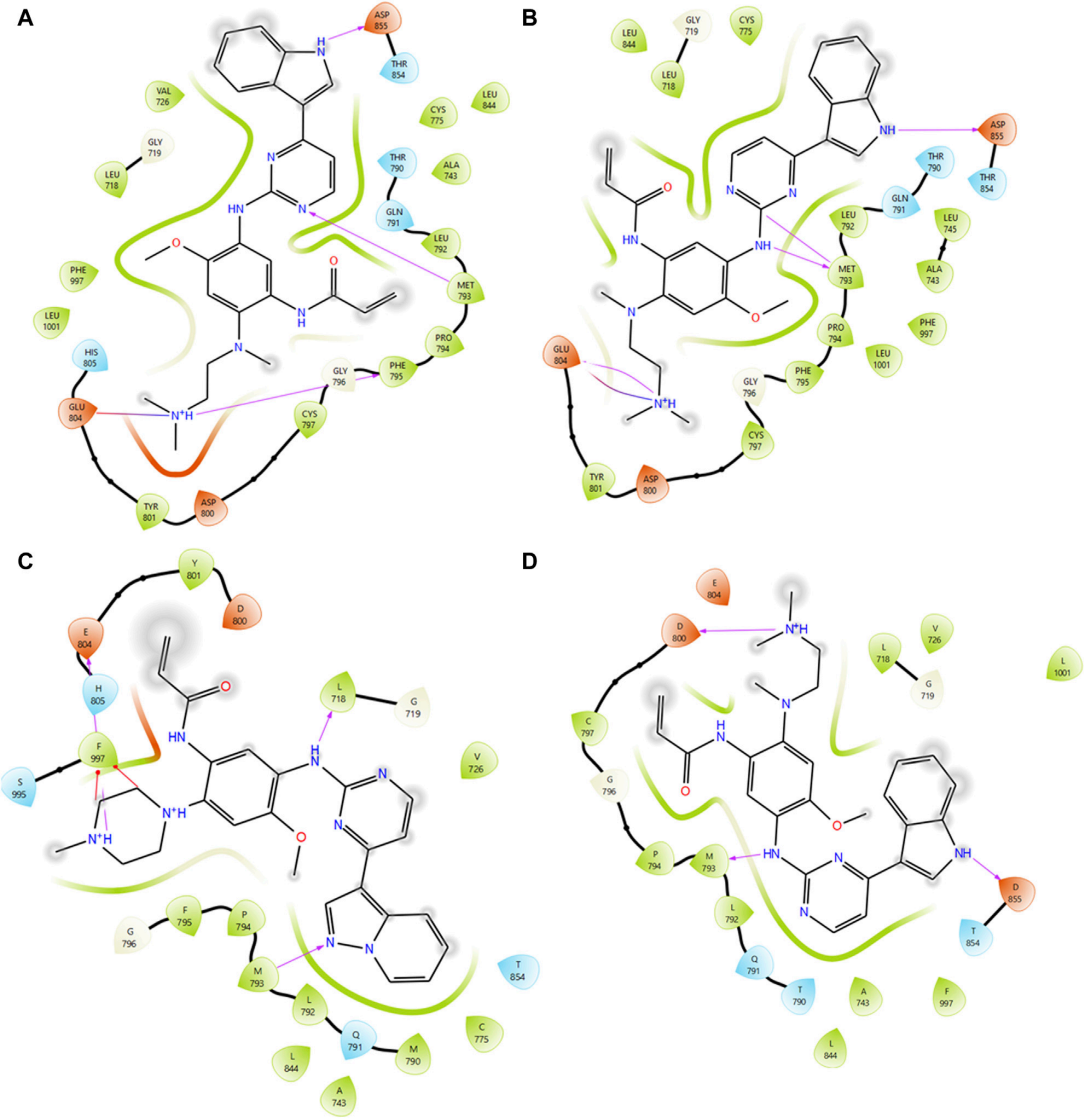
Interaction analysis between mutant type EGFRs with virtually screened compounds. **(A)** Interaction analysis between G724E with CID:71496460. **(B)** Interaction analysis between K745L with CID:71496460. **(C)** Interaction analysis between mutant T790M/L858R with CID 73292362. **(D)** Interaction analysis between mutant V851D with CID 73292545.

Virtual screening plays a pivotal role in identifying potential small molecules by systematically screening chemical libraries for compounds that can bind to a target protein (Cuccioloni et al., 2020). In this study, we screened a total of 7385 chemical compounds obtained from the PubChem and DrugBank databases against the mutant EGFR structures (G724E, K745L, V851D, and T790M/L858R) using the *Schrodinger Glide* virtual screening workflow. The top ten compounds were selected for each mutant structure based on their binding energies and the number of hydrogen bond interactions with the mutant EGFR structures. Subsequently, independent docking analyses were performed for each compound against each mutant structure to identify three compounds (CID 71496460, 73292362, and 73292545) that show the potential to selectively inhibit EGFR despite drug-resistance mutations (Supplementary Figure S5). These compounds were selected based on their favorable binding energies and ability to form higher number of hydrogen bonds with the mutant proteins as shown in Table 3. For instance, compound CID 71496460 exhibited a favorable binding energy of −8.376 kcal/mol and formed three hydrogen bonds with the mutant G724E structure at residues PHE795, MET793, and ASP855 (Figure 4A). This compound also demonstrated a promising binding energy of −8.002 kcal/mol and formed four hydrogen bonds with the EGFR mutant, K745L, at residues MET793, GLU804, and ASP855 (Figure 4B). Similarly, CID 73292362 displayed an intense binding energy of −9.110 kcal/mol with the double mutant, T790M/L858R (Figure 4C) and CID 73292545 exhibited a considerable binding energy (−7.649 kcal/mol) with V851D (Figure 4D).

**TABLE 3:** Molecular docking between virtually screened best compounds with their respective mutant structures shows binding energy, hydrogen bond number, and the amino acids involved in hydrogen bond formation.

Mutant EGFR’s	PubChem ID	XP gscore (Kcal/mol)	Number of hydrogen bonds between the target & compound	Amino acids involved in hydrogen bond formations
G724E	71496460	−8.376	3	PHE795, MET793, ASP855
K745L	71496460	−8.002	4	MET793, GLU804, ASP855
T790M/L858R	73292362	−9.110	3	LEU718, MET793, ASP804
V851D	73292545	−7.649	3	MET793, ASP800, ASP855

### 3.5 ADME assessment highlights the promise of the screened drug candidates

*QikProp*, a computational tool, provides valuable predictions on important molecular descriptors and pharmaceutical properties of organic compounds. The ADME (Absorption, Distribution, Metabolism, and Excretion) profile, which assesses the drug-like behavior of a chemical agent, was evaluated for the three compounds and the results were presented in Table 4. Notably, none of the screened compounds violated the Lipinski rule criteria, as indicated by a star value of zero. The star rating system, ranging from 0 to 5, suggests that compounds with fewer stars possess more extraordinary drug-like characteristics. Additionally, the molecular weight, number of H-bond donors and acceptors, and logP values of the screened compounds fall within the acceptable ranges defined by the Lipinski rule. Based on these favorable properties, these three compounds merit consideration for further investigation.

**TABLE 4:** ADME analysis for the screened lead compounds displayed along with the screened compound molecular properties.

Screened lead molecules	Stars	Molecular weight (Dalton)	Hydrogen bond donor	Hydrogen bond acceptor	Log-*p*-value
71496460	0	485.588	3	8	4
73292362	0	484.56	2	9	3
73292545	0	483.572	3	8	4

### 3.6 MD simulation reveals higher binding efficacies between the screened compounds and mutant EGFR structures

**FIGURE 5:**
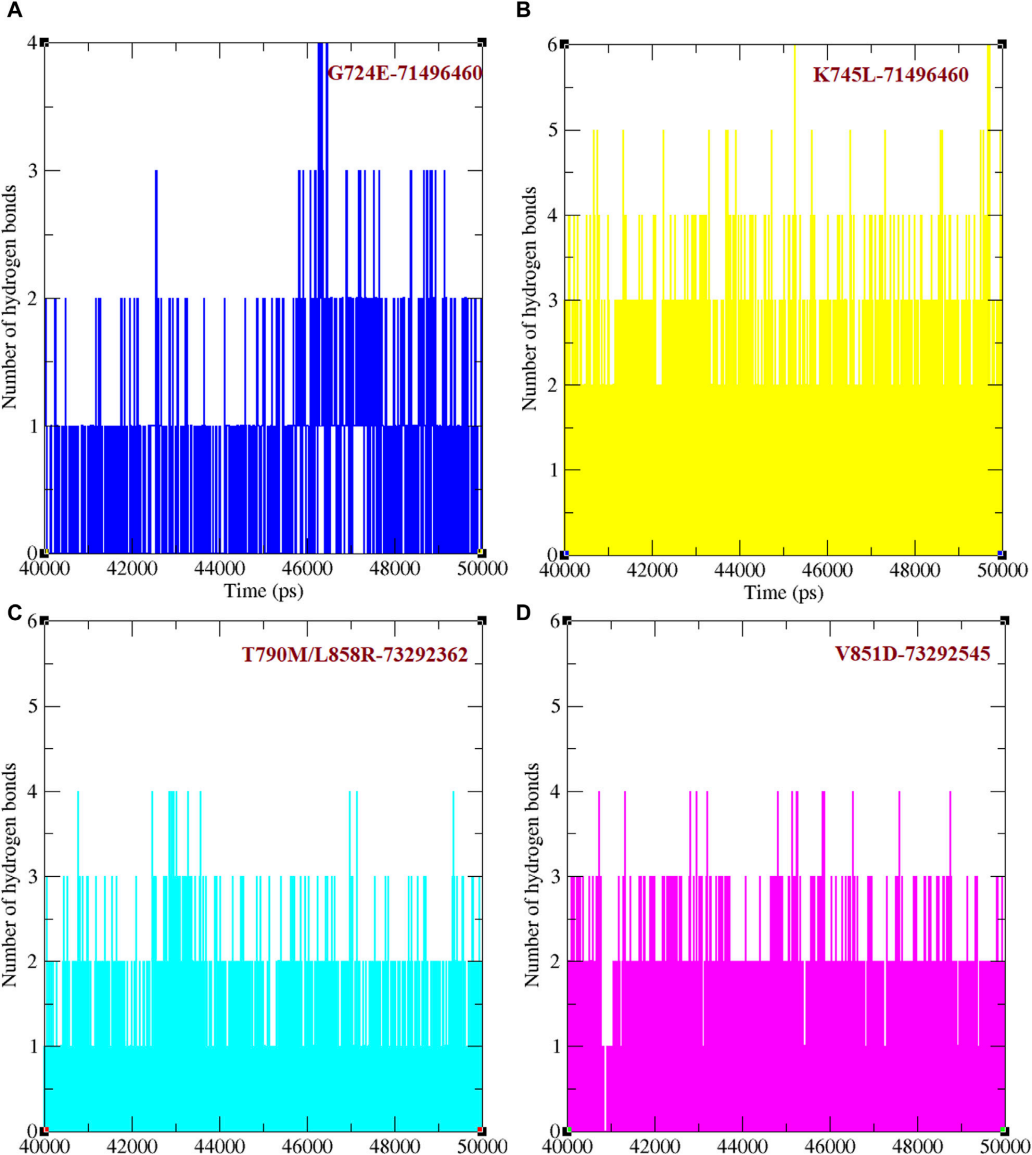
Total number of hydrogen bonds formed between mutant EGFRs with its respectively screened compounds. **(A)** The number of hydrogen bonds formed between G724E-71496460. **(B)** The number of hydrogen bonds formed between K745L-71496460. **(C)** The number of hydrogen bonds formed between T790M/L858R-732992362. **(D)** The number of hydrogen bonds formed between V851D-73292545.

**FIGURE 6:**
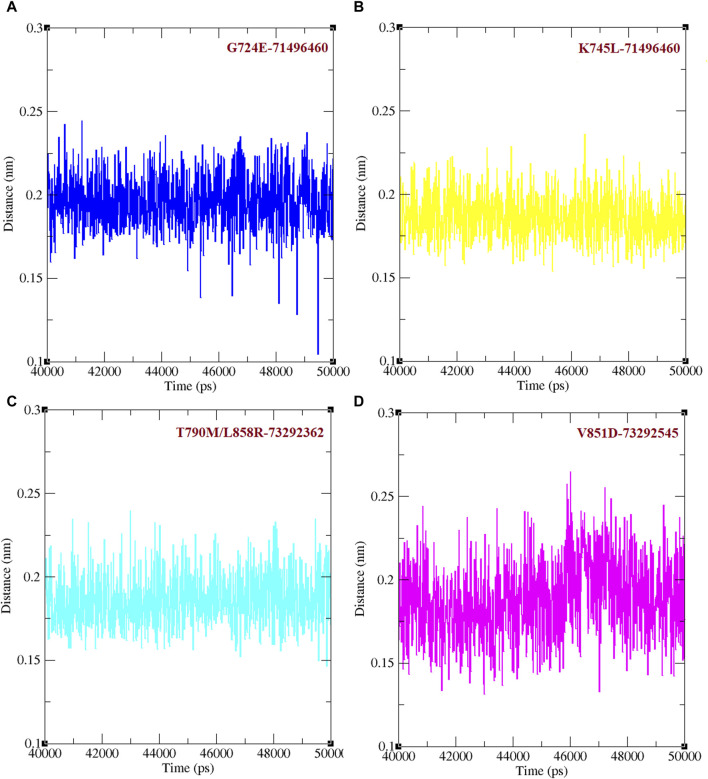
Minimum distance between mutant EGFR with its respectively screened compounds. **(A)** The minimum distance between G724E-71496460. **(B)** The minimum distance between K745L-71496460. **(C)** The minimum distance between T790M/L858R-732992362. **(D)** The minimum distance between V851D-73292545.

Binding efficacies were evaluated based on both the distance between the compound and protein structure and the number hydrogen bonds between them using a 50 ns MD simulation (Figure 5). In the last 10 ns of the simulation, G724E-71496460, K745L-71496460, T790M/L858R-732992362, and V851D-73292545 have maintained approximately 0–4, 0–6, 0–4, and 0–4 hydrogen bonds, respectively. Notably, compared to the FDA-approved drugs, osimertinib and afatinib, all the screened compounds exhibited higher number of hydrogen bonds during the MD simulation period. Similarly, in the last 10 ns of the simulation period, G724E-71496460, K745L-71496460, T790M/L858R-732992362, and V851D-73292545 maintained minimum distance ranges of approximately 0–0.25 nm, 0.15–0.27 nm, 0.15–0.22 nm, and 0.15–0.25 nm, respectively (Figure 6). Again, compared to the FDA-approved drugs, all the screened compounds exhibited shorter distances with corresponding mutant structures, indicating their potential to inhibit the EGFR mutant proteins more effectively.

## 4 Discussion

EGFR plays a critical role in the development and progression of various cancers (Inamura et al., 2010). It is a cell surface receptor belonging to the receptor tyrosine kinase (RTK) family, involved in regulating cell growth, proliferation, and survival (Ohsaki et al., 2000). Dysregulation of EGFR signaling has been implicated in multiple cancer types, making it an attractive target for cancer therapy (Guardiola et al., 2019). EGFR overexpression is observed in a significant subset of colorectal cancers and elevated EGFR signaling is associated with enhanced tumor growth and metastasis (Oh et al., 2011). Anti-EGFR monoclonal antibodies like cetuximab and panitumumab have been developed to target EGFR in colorectal cancer, particularly in patients with wild-type RAS status (Karapetis et al., 2008). These therapies have shown clinical benefit in patients with EGFR overexpressing tumors. EGFR amplification and mutations are frequent events in Glioblastoma Multiforme (GBM) (Marvalim et al., 2023). EGFRvIII, a constitutively active EGFR variant, is commonly observed in GBM and associated with a more aggressive phenotype. Targeting EGFR signaling in GBM has been challenging, but various approaches, including EGFR-specific TKIs and monoclonal antibodies, are being investigated in clinical trials (Marvalim et al., 2023). EGFR is frequently overexpressed and activated in Head and Neck Squamous Cell Carcinoma (HNSCC) (Vermorken et al., 2008). This overexpression is associated with poor prognosis and resistance to conventional therapies. EGFR-targeted therapies, such as cetuximab, have been approved for the treatment of recurrent or metastatic HNSCC, improving patient outcomes (Bonner et al., 2006). EGFR mutations are prevalent in NSCLC, particularly in adenocarcinoma. These mutations lead to constitutive activation of the EGFR pathway, promoting uncontrolled cell growth and cancer development. EGFR TKIs like gefitinib, erlotinib, and osimertinib have been developed to target these mutations in NSCLC (Paez et al., 2004). Osimertinib, a third-line drug, is specifically designed to overcome EGFR resistance that arises after treatment with second-like TKIs like afatinib. Nevertheless, despite the remarkable clinical efficacy of osimertinib, patients inevitably develop acquired resistance, posing a significant challenge due to the limited availability of post-osimertinib pharmacological options.

This study utilizes a combination of sequence data analysis, molecular docking, virtual screening, and molecular dynamics simulation to detect drug resistance mutations in NSCLC patients and identify three compounds that show promise to inhibit EGFR mutant proteins. We identified five EGFR-specific somatic mutations within the WES dataset that include five single-point (L718A, G724E, G724K, K745L, V851D) and one double (T790M/L858R) mutations associated with drug resistance. All these mutations are activating, causing the EGFR protein to become hyperactive, leading to uncontrolled cell growth and division in NSCLC patients (Lv et al., 2020). L718A, the most common EGFR mutation found in approximately 40% of NSCLC patients, substitutes leucine with alanine at position 718 (Zhang et al., 2019). This change alters the way the EGFR protein interacts with drugs. Leucine is a hydrophobic amino acid located in the extracellular domain of the EGFR protein, anchoring it to the cell membrane. In contrast, alanine is also hydrophobic but smaller, potentially affecting interactions with other molecules due to its smaller size.

The less common mutations, G724E, G724K, K745L, and V851D, were found in 1%–10% of NSCLC patients (Tu et al., 2017; Brindel et al., 2020). Both G724E and G724K mutations replace glycine at position 724 with glutamic acid and lysine, respectively, the G724 mutations leading to increased EGFR activity and responsiveness to Epidermal Growth Factor (EGF) (Oztan et al., 2017). These mutations are in the EGFR protein’s extracellular domain that is responsible for drug molecule binding (Brindel et al., 2020). The K745L mutation alters the amino acid at position 745, where lysine plays a role in anchoring the EGFR protein to the cell membrane and activating it when bound to EGF (Bean et al., 2008). Lysine is a larger than leucine and a positively charged amino acid and this substitution can change the shape and solvent accessibility of the EGFR protein, which can affect its ability to interact with other molecules. The double mutation, T790M/L858R, occurs in exon 20 of the EGFR gene (Fujiwara et al., 2020). T790M mutation replaces threonine with methionine, while L858R replaces lysine with arginine. Both these mutations have the potential to alter EGFR’s drug interactions as the substituting amino acids could affect the polarity and charge properties of the protein potentially obstructing drug binding.

To the best of our knowledge, this study is the first to report the V851D mutation in EGFR as drug resistant in NSCLC cases. Substitution of aspartic acid for valine at position 851 in the V851D mutant imparts additional negative charge potentially altering its folding as well as function. Moreover, position 851 falls in the tyrosine kinase domain of EGFR, which is responsible for binding to other molecules, and this mutation could negatively affect its binding to other proteins or drugs due to altered electrostatic interactions within the protein.

Among the six mutant structures considered for molecular docking analysis, K745L and V851D exhibited high binding energies and a slightly modified binding orientation when interacting with osimertinib. Similarly, G724E and T790M/L858R also showed high binding energy and a slightly modified orientation when interacting with afatinib. The higher the binding energy, the lower the binding affinity, and *vice versa*. Molecular docking analysis also elucidated mutations that impact the drug-binding abilities of EGFR as indicated by the elevated RMSD values due to significant conformational changes in the mutant EGFR proteins. Hydrogen bond analysis revealed fewer hydrogen bonds formed between the mutant structures with corresponding drugs compared to WT EGFR. Similarly, in the minimum distance analysis, it was observed that the mutant structures exhibited greater distances compared to WT EGFR when interacting with afatinib or osimertinib. The hydrogen bond and minimum distance analyses confirmed that all drug resistance mutations affected the conformation of the drug-binding pocket, consequently disrupting the usual non-bonded interactions with afatinib and osimertinib. The SASA measurement, reflecting the overall surface area of the protein structure, indicated the potential interaction areas with other molecules. WT EGFR exhibited a larger surface area than the mutant structures, indicating that drug-resistant mutations rendered the EGFR structures more compact. Collectively, these findings contribute to a comprehensive characterization of the WT and mutant complexes and their implications for drug binding.

Our next goal is to identify new drug compounds that could potentially inhibit the mutant EGFR activity. Using virtual screening, we screened for compounds that have structural similarity to FDA-approved afatinib and osimertinib to determine the most effective compound to block the mutant EGFR structures. A total of 7385 chemical compounds were screened against four mutant EGFR structures (G724E, K745L, V851D, and T790M/L858R) resulting in the identification of three compounds (CID 71496460, 73292362, and 73292545) that exhibited strong binding affinity with the EGFR mutant structures. CID 71496460 was identified to target both G724E and K745L mutants, while CID 73292362 and CID 73292545 were found to be good candidates for T790M/L858R and V851D mutants, respectively. These compounds demonstrated similar characteristics to osimertinib and established more hydrogen bonds with the mutant EGFR than afatinib and osimertinib. In this context, the drug resistance mutations within the binding site induced subtle conformational changes that affected the binding of afatinib and osimertinib. Conversely, compounds similar to these drugs might possess slight conformational variations that enable them to fit the newly acquired conformation of EGFR resulting from the mutations. Molecular dynamic simulations were performed to delve deeper into the efficacy of the screened compounds, analyzing crucial parameters such as hydrogen bond formation and minimum distance analysis. The analysis of hydrogen bonds revealed that the screened compounds formed more hydrogen bonds than the original drugs, while the minimum distance analysis demonstrated that the identified compounds exhibited reduced distances relative to the approved drugs. These analyses further confirm the suitability of using the three screened compounds as effective inhibitors of the mutant EGFR proteins, which should be further evaluated by experimental studies.

## 5 Conclusion

EGFR inhibitors have revolutionized cancer treatment, offering substantial benefits in managing various malignancies. However, the intricate nature of tumor biology, marked by heterogeneity and genomic instability, poses a significant challenge in the form of anticancer drug resistance, particularly with EGFR inhibitors. Our research has identified specific drug resistance mutations using WES data that hyperactivate the EGFR protein, leading to uncontrolled cell proliferation in NSCLC patients. This resistance to anti-cancer drugs highlights the urgent need for alternative approaches to effectively combat drug resistance in the EGFR-driven tumors. Through virtual screening, we have successfully identified lead compounds with the potential to inhibit EGFR activity in the presence of identified drug resistance mutations. This promising avenue offers hope for developing effective and personalized treatment options for patients with heterogeneous genetic backgrounds. By targeting drug-resistant EGFR mutations and leveraging the potential of NGS technologies, we aim to pave the way for more personalized and effective treatments, ultimately improving outcomes and quality of life for those affected by EGFR-driven NSCLC cancers.

## Data Availability

Publicly available datasets were analyzed in this study. This data can be found here: SRA ID PRJEB21459, PRJNA616048/dbGaP: phs002001, PDB ID: 3VJO.

## Supplementary material

Supplementary Figure S1 Interaction analysis between WT and mutant type with drug osimertinib. **(A)** Interaction analysis between wild-type EGFR with osimertinib. **(B)** Interaction analysis between G724K with osimertinib. **(C)**. Interaction analysis between mutant K745L with osimertinib. **(D)** Interaction analysis between mutant V851D with osimertinib.

Supplementary Figure S2 Interaction analysis between WT and mutant type with drug afatinib. **(A)** Interaction analysis between wild-type EGFR with afatinib. **(B)**. Interaction analysis between L718A with afatinib. **(C)** Interaction analysis between mutant G724E with afatinib. **(D)** Interaction analysis between mutant T790M/L858R with afatinib.

Supplementary Figure S3 Root mean square deviation analysis for WT and mutant EGFR structures interacts with **(A)** Osimertinib and **(B)** Afatinib.

Supplementary Figure S4 Solvent accessible surface analysis for WT and mutant EGFR structures interacts with **(A)** Osimertinib and **(B)** Afatinib.

Supplementary Figure S5 Virtually screened compounds against EGFR mutant structures: **(A)** CID:71496460, **(B)** CID:73292362, and **(C)** CID: 73292545.
