# Supplementary material for: Identification of potential inhibitors for drug-resistant EGFR mutations in non-small cell lung cancer using whole exome sequencing data
Source: Front Pharmacol. 2024 Jul 25;15:1428158. doi: 10.3389/fphar.2024.1428158 (PMC11310931; doi:10.3389/fphar.2024.1428158)
Supplement: Supplementary file 1 [file DataSheet1.docx]

**Supplementary Figure S1**

**
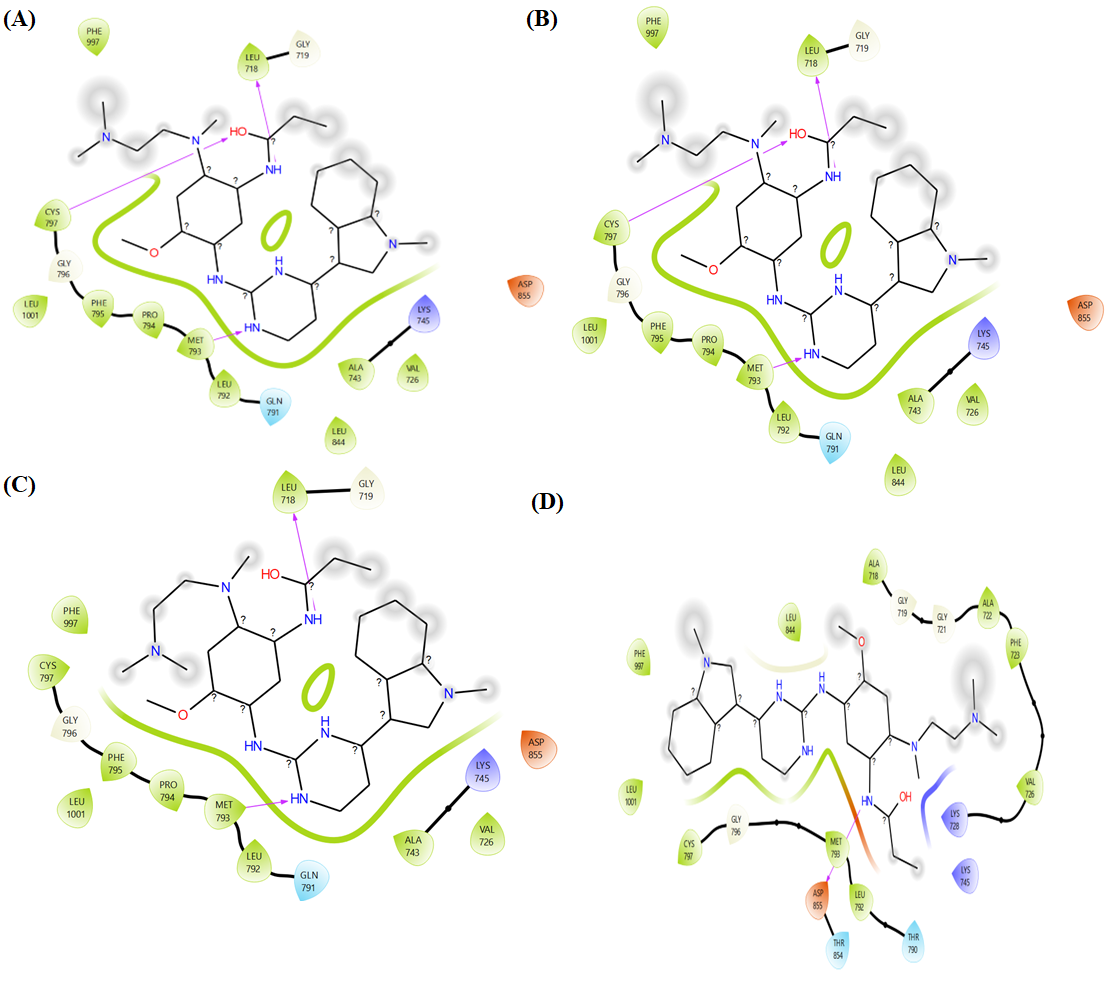
Supplementary Figure S2**

**
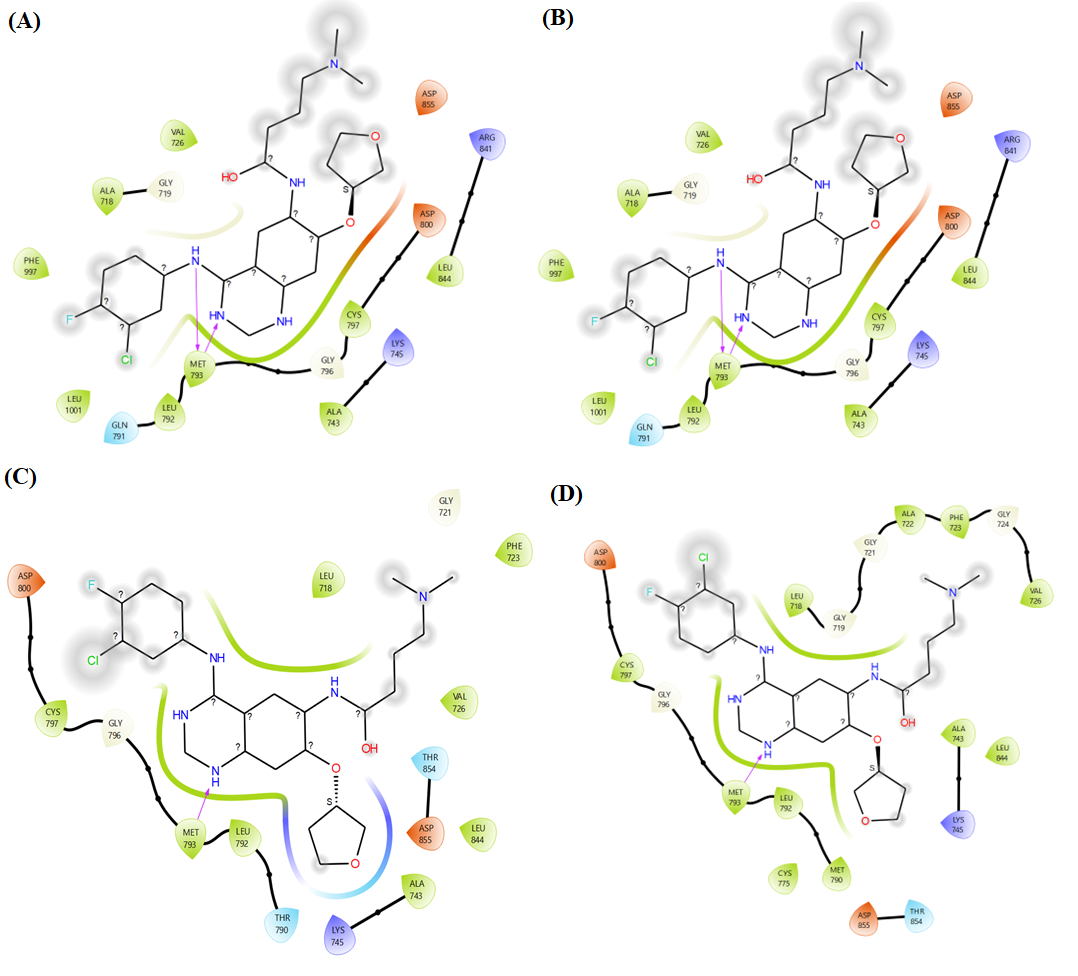
 Supplementary Figure S3**

**
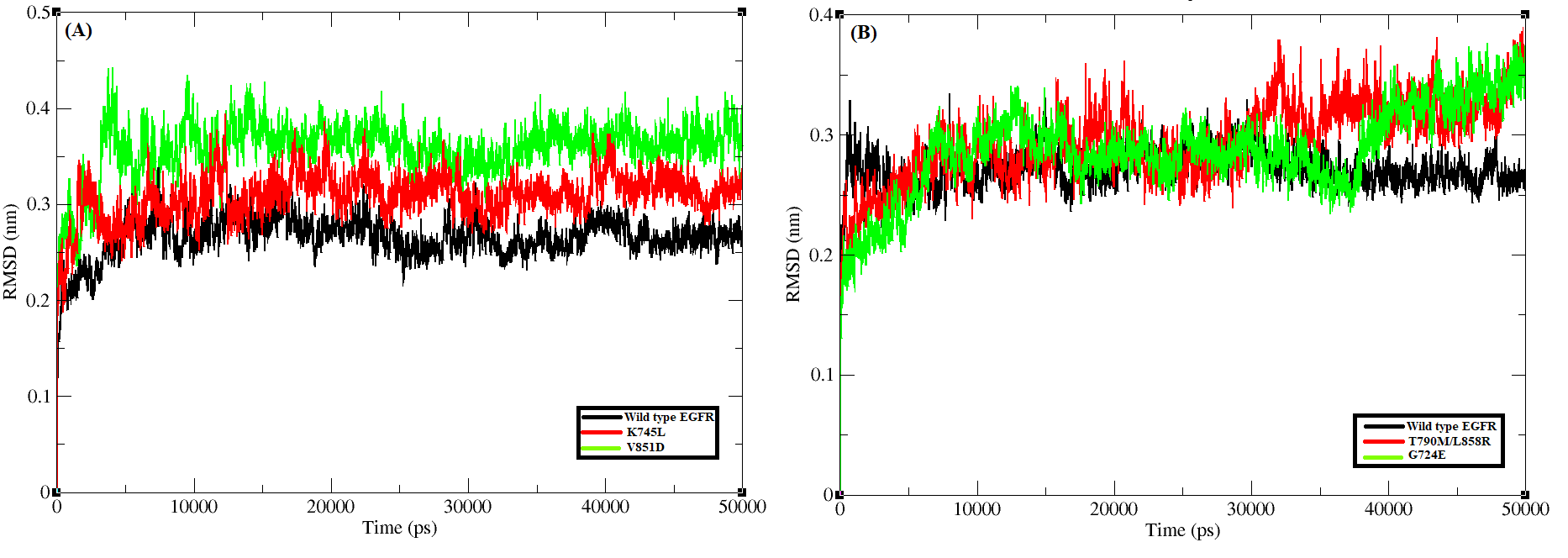
 Supplementary Figure S4**

**
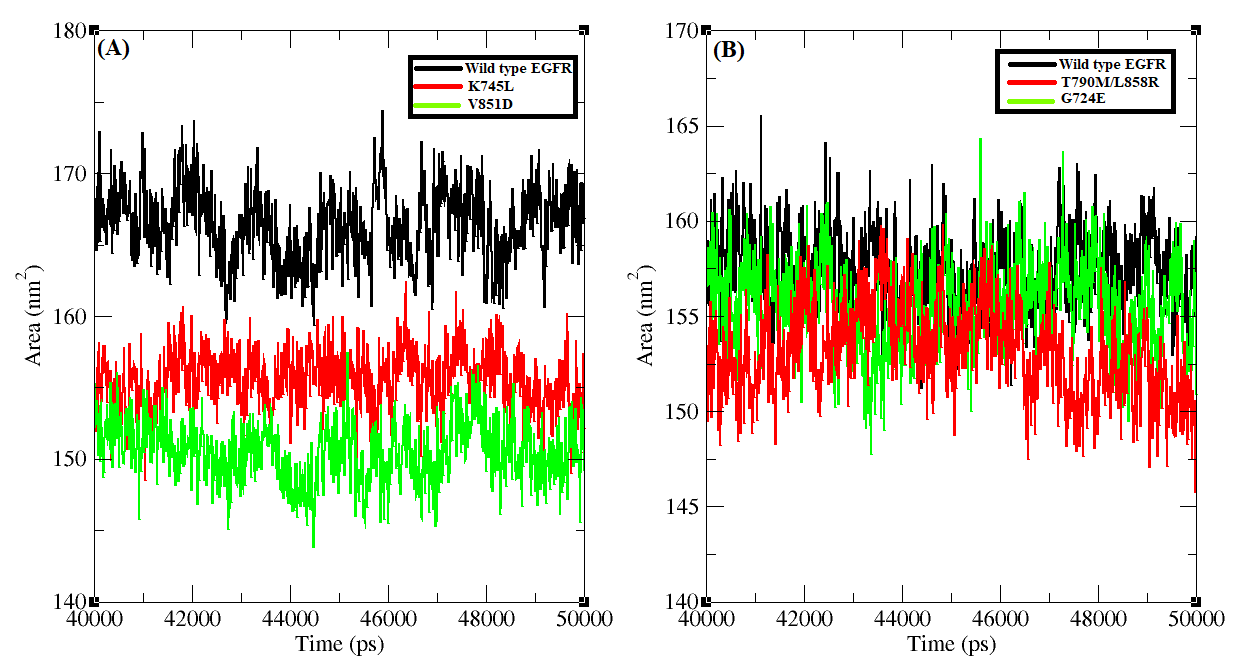
 Supplementary Figure S5**

**
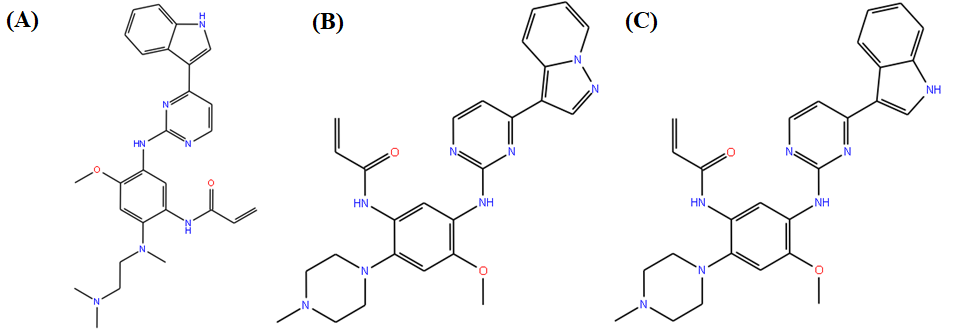
**
